# Comparative assessment of functional groups and surface morphology of dental and peri-implant calculus: A cross-sectional study

**DOI:** 10.34172/japid.026.3931

**Published:** 2026-05-24

**Authors:** Behzad Houshmand, Anahita Moscowchi, Helia Faeli, Parsa Dayani

**Affiliations:** ^1^Department of Periodontics, School of Dentistry, Shahid Beheshti University of Medical Sciences, Tehran, Iran; ^2^Dental Research Center, Research Institute for Dental Sciences, Shahid Beheshti University of Medical Sciences, Tehran, Iran

**Keywords:** Dental calculus, Dental implants, Electron microscopy, Fourier-transform infrared spectroscopy

## Abstract

**Introduction:**

This research aimed to compare functional groups and surface morphology of dental and peri-implant calculus.

**Methods:**

Six patients with dental implants in the anterior lower jaw were included. Calculus samples were collected from the implant surfaces and the corresponding contralateral natural teeth. Fourier-transform infrared spectroscopy (FTIR) and field emission scanning electron microscopy (FESEM) analyses were performed.

**Results:**

Distinct differences were observed between dental and peri-implant calculus in both functional groups and surface structure. FTIR analysis revealed higher intensity of oxygen-related bond peaks around 3426–3428 cm^-1^ in implant calculus, along with increased signals for C=C stretching near 1646–1649 cm^-1^, suggesting variations in the organic matrix. Additional peaks at 2919–2921 cm^-1^ and 1451–1456 cm^-1^ were present in both groups, indicating aliphatic C–H bonds. FESEM imaging revealed that dental calculus had a compact surface, dense microbial plaque, and minimal porosity. In contrast, peri-implant calculus displayed a layered architecture with multiple pores ranging from approximately 1–3 µm in diameter. These morphological differences suggest divergent mineralization patterns and biofilm retention mechanisms.

**Conclusion:**

These differences suggest distinct mechanisms of plaque retention and mineralization in peri-implant versus natural tooth environments, potentially influenced by environmental and biological factors. These findings may offer insights that inform preventive strategies and ultimately improve implant longevity.

## Graphical Abstract

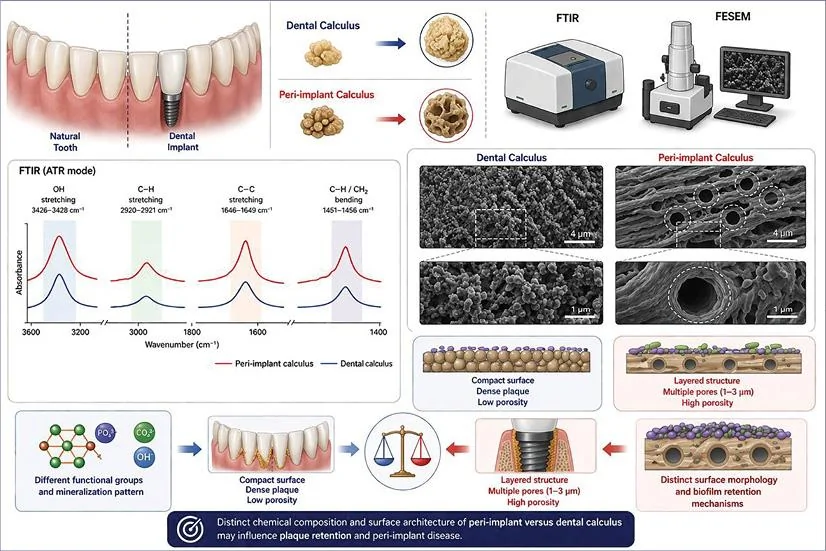

## Introduction

 Dental calculus is composed of mineral substances and an organic matrix, essentially representing mineralized plaque.^1^ Microbial plaque is the primary initiator of periodontal and peri-implant diseases,^2,3^ and since calculus is the most significant local factor in plaque retention, it can play a major role in the occurrence of these complications. Mucositis is limited to the soft mucosal tissues and does not cause bone resorption,^4^ whereas peri-implantitis is a destructive inflammation that causes progressive bone resorption around a dental implant.^2^ With the progression of inflammation around the implant and the spread of bacteria, peri-implantitis develops, causing complications similar to those associated with the periodontium of natural teeth and may even lead to implant failure.^5^

 Recent evidence suggests that biofilm accumulation and calculus contribute to peri-implant disease pathogenesis, as they do in periodontitis.^6^ Extending this concept to implants, peri-implant calculus may act as a persistent nidus for bacterial colonization, thereby triggering or sustaining peri-implant inflammation. Recent studies have also revealed differences in the composition and maturation of biofilms on titanium and zirconia surfaces compared with enamel,^7,8^ raising important questions about their influence on mineralization patterns and clinical outcomes. The mineralization process of microbial plaque involves the gradual conversion of brushite into octacalcium phosphate and whitlockite, and then into more stable hydroxyapatite structures. Experiments have shown that there is little difference between the crystal size of calculus in anterior and posterior teeth in the same jaw. However, the amount of calcium and phosphate differs between the two jaws.^1^

 Although several studies have compared the microbial and biochemical composition of dental and implant plaque,^7,9^ to the best of the authors’ knowledge, no prior study has directly compared the chemical composition and surface morphology of dental versus peri-implant calculus. Functional groups such as phosphate, carbonate, and hydroxyl contribute to the mineral structure and bonding strength of calculus, and their distribution may influence the tenacity and removability of deposits. Additionally, the surface energy and oxide layer properties of implant materials can affect ion adsorption and crystal orientation, potentially altering the extent and texture of mineralization.^10,11^

 Therefore, this study aimed to assess and compare the functional groups and surface morphology of calculus formed around dental implants and natural teeth. We hypothesized that peri-implant calculus exhibits distinct compositional and structural characteristics compared to dental calculus, reflecting differences in mineralization pathways and biofilm retention mechanisms.

## Methods

 This study was conducted in accordance with the Declaration of Helsinki and approved by the Ethics Committee of Shahid Beheshti University of Medical Sciences, Tehran, Iran (Approval ID: IR.SBMU.DRC.REC.1402.029). Written informed consent was obtained from each participant before enrollment.

###  Inclusion Criteria

 Participants were eligible if they met the following conditions:

- Age between 18 and 68 years
- Availability of probing depth, clinical attachment level, and bleeding on probing
- No history of periodontal therapy within the preceding six months
- At least 12-month of functional loading

###  Exclusion Criteria

 The participants were excluded based on the following conditions:

- Current smokers or individuals who had quit smoking within the past 12 months
- Presence of systemic conditions known to influence calculus formation, including diabetes mellitus, autoimmune diseases (e.g., Sjögren’s syndrome), or salivary gland dysfunction
- Use of medications affecting salivary flow or composition, such as anticholinergics or calcium channel blockers
- Pregnancy or lactation at the time of enrollment

These criteria were applied to ensure a homogenous study population and to minimize confounding factors related to periodontal health and calculus formation.

 Calculus samples were collected from implants and corresponding contralateral natural teeth. While examining the implants before collecting calculus samples, mild bleeding on probing was observed; however, no progressive bone resorption was identified surrounding the implants at the time of evaluation, indicating a diagnosis of peri-implant mucositis. All samples were taken from supragingival calculus and rinsed with deionized water to remove residual debris.

 The specimens were subjected to a graded ethanol dehydration protocol using sequential concentrations of 30%, 50%, 70%, 90%, and 100% for 10 minutes each. Following dehydration, the specimens were dried in a vacuum desiccator and subsequently sputter-coated with a ~10-nm gold layer using a Quorum Technologies SC7620 sputter coater. Morphological analysis was performed using a field emission scanning electron microscope (FESEM; MIRA3, TESCAN, Brno, Czech Republic) operated at 25 kV with a working distance of 5 mm. Images were acquired at magnifications ranging from 1000 × to 10,000 × to evaluate the surface morphology of dental calculus.

 Fourier-transform infrared spectroscopy (FTIR) spectra were collected using an AVATAR spectrometer (Thermo Fisher Scientific, Waltham, MA, USA) equipped with a Specac Golden Gate attenuated total reflectance (ATR) accessory featuring a diamond crystal. Each spectrum was acquired with 64 scans at a resolution of 4 cm - ¹. Baseline correction was applied using the instrument’s built-in automatic polynomial fitting algorithm. Peak intensities were quantified using OriginPro 2023 (OriginLab Corp., Northampton, MA, USA) via Gaussian peak fitting and area integration. FTIR instrument calibration was verified using a standard potassium bromide (KBr) reference before sample analysis. Spectral reproducibility and baseline stability were confirmed through repeated scans of the control.

 All compositional and morphological comparisons were descriptive; no statistical analyses were conducted.

## Results

 Six patients (4 males and 2 females) with one dental implant in the anterior mandible were selected for this study. Patients aged 44‒68 years, with a median age of 49.5 years.

 FTIR spectroscopy revealed distinct compositional differences between peri-implant and dental calculus samples. Both spectra exhibited characteristic phosphate (PO₄^3-^) stretching vibrations near 1030–1085 cm^-1^ and carbonate (CO₃^2-^) bending around 874 cm^-1^, confirming the presence of mineralized components such as hydroxyapatite. However, peri-implant calculus showed markedly stronger absorption bands at 1646–1649 cm^-1^ and 1542 cm^-1^, corresponding to C = C stretching and amide I/II vibrations, respectively. These peaks suggest a higher content of unsaturated organic compounds and proteinaceous biofilm matrix. Additionally, broader O–H and C–H stretching bands around 3426 and 2919 cm^-1^ were observed in peri-implant samples, indicating increased hydration and lipid content. In contrast, dental calculus exhibited sharper, more defined mineral peaks, consistent with greater crystallinity and a lower organic-to-mineral ratio (Table 1). Figures 1 and 2 present the FT-IR analysis of peri-implant and dental calculus, respectively.

**Table 1:** Key functional groups identified by Fourier-transform infrared spectroscopy analysis in the study samples

**Wave number (cm^-1^)**	**Functional Group**	**Assignment**	**Observations**
~3426-3428	O–H stretching	Water, hydroxyl groups	Present in both; broader in peri-implant calculus, suggesting a more hydrated organic matrix
~2919-2921	C–H stretching	Aliphatic hydrocarbons	Slightly stronger in peri-implant calculus, indicating higher lipid content
~1646-1649	C = C / Amide I	Unsaturated compounds/proteins	More intense in peri-implant calculus, reflecting microbial biofilm and EPS
~1542	Amide II	Protein backbone (N–H bending)	Elevated in peri-implant calculus, consistent with bacterial protein presence
~1451-1456	CH_2_ bending	Lipid chains	Present in both, more pronounced in peri-implant samples
~1031-1084	PO_4_^3-^ stretching	Phosphate groups (apatite)	Strong in both, slightly sharper in dental calculus, indicating higher crystallinity
~874	CO_3_^2-^ bending	Carbonate substitution	Present in both; more diffuse in peri-implant calculus
~602-669	PO_4_^3-^ bending	Crystalline phosphate	Stronger in dental calculus, suggesting a more mature mineral phase

**Figure 1:**
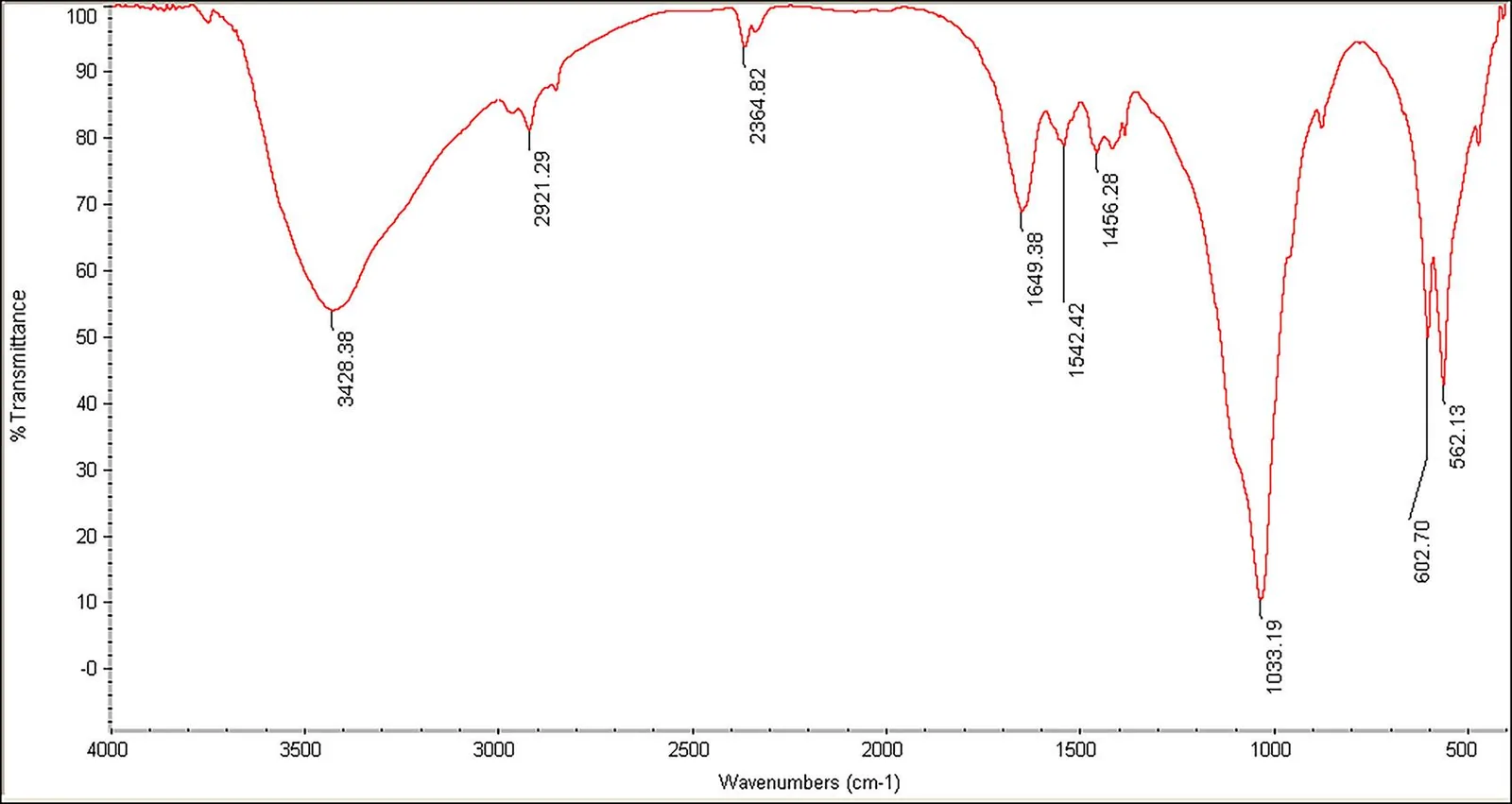
Representative Fourier Transform Infrared Spectroscopy spectra of peri-implant calculus sample. The diagram illustrates the infrared absorption bands corresponding to key functional groups identified in the samples

**Figure 2:**
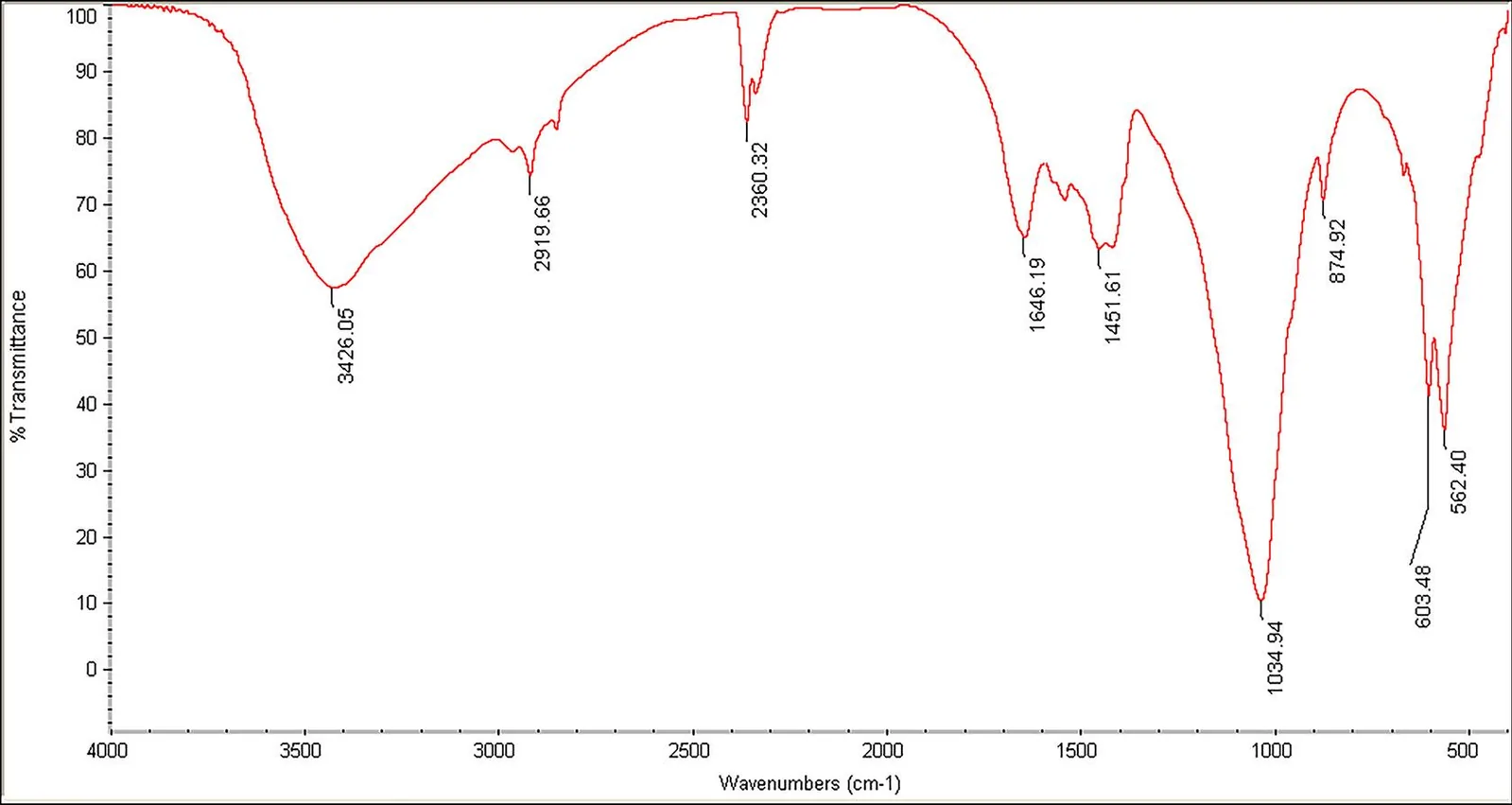
Representative Fourier Transform Infrared Spectroscopy spectra of a dental calculus sample. The diagram illustrates the infrared absorption bands corresponding to key functional groups identified in the samples

 The FESEM imaging results revealed that dental calculus exhibited a higher accumulation of microbial plaque with densely packed coccoid and short-rod bacterial imprints measuring approximately 0.5–1.0 µm in diameter. The surface texture was dense with limited visible pores (< 1 µm), indicative of a robust biofilm matrix. In contrast, implant calculus demonstrated a layered structure with multiple pores (range: ~1–3 µm in diameter) in certain regions and microchannels (~0.4–0.7 µm wide) traversing between lamellae. Additionally, the surface of implant calculus contained a lower proportion of non-mineralized material than dental calculus, suggesting differences in the maturation or mineralization processes between the two types of samples. Figures 3 and 4 illustrate the FESEM morphological analysis of peri-implant and dental calculus, respectively.

**Figure 3:**
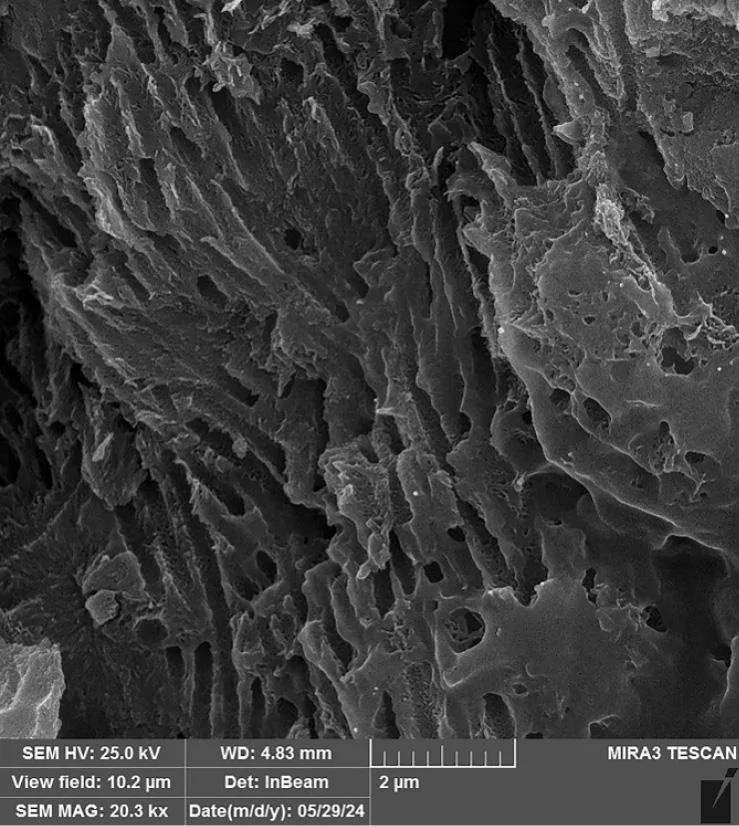
Scanning electron micrograph of peri-implant calculus at × 20,000 magnification. The image reveals the microstructural complexity of peri-implant calculus, characterized by irregular mineralized deposits, porous architecture, and fractured surface features

**Figure 4:**
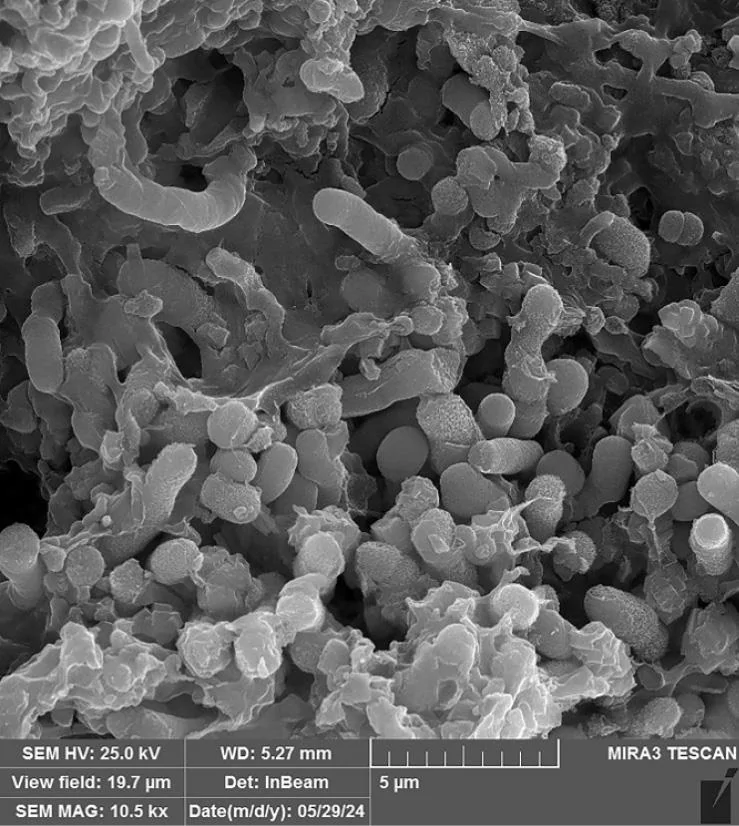
Scanning electron micrograph of dental calculus at × 10,000 magnification. The image reveals a dense microbial biofilm embedded within the mineralized matrix of dental calculus. Rod-shaped and coccoid bacteria are visible across the irregular surface, suggesting mature colonization and extracellular matrix formation

## Discussion

 Our findings revealed some differences in the functional groups and surface morphology between dental and implant calculus. It has been documented that biofilm development on dental implants and natural teeth follows a similar pattern. Nonetheless, certain distinctions arise from the materials used in the implants. These materials exhibit reduced albumin adsorption compared with natural teeth. Consequently, this characteristic leads to reduced biofilm formation around the implants. Biofilm formation initiates on the implant surface approximately 30 minutes after exposure to the oral environment. In contrast, biofilms associated with natural teeth form within minutes.^6^ This is consistent with the findings of the present study, as the biofilm was denser on dental calculus. A denser plaque layer may promote greater organic matrix retention and more advanced mineralization, potentially influencing the chemical composition detected in FTIR spectra. Therefore, analyzing variations in functional groups can provide insights into how biofilm density affects the balance between organic and inorganic components during calculus formation.

 Infrared (IR) spectroscopy is a widely used method for identifying functional groups and chemical compounds, encompassing both organic and inorganic substances, as well as evaluating the purity of a compound.^12^ This technique is cost-effective, the instruments are user-friendly, and IR spectra can be rapidly acquired. Infrared spectroscopy is a promising alternative to other methodologies, as it does not require extensive time for sample preparation, measurement, or result interpretation; furthermore, it is non-invasive and relatively straightforward to implement. In FT-IR spectroscopy, all frequencies are measured simultaneously, allowing the spectrum to be generated within a few seconds, which is advantageous compared to traditional IR spectroscopy, which can be time-consuming. A significant benefit of IR spectroscopy techniques is their ability to analyze small quantities of material, and, in the case of the ATR technique, the light beam can penetrate the sample to a depth of approximately 0.5–3 µm. The benefit of ATR analysis is that it enables microanalysis in situ with little or no preparation.^12^

 FT-IR analysis of the samples in this study revealed that implant calculus exhibited a higher intensity of oxygen bond peaks at 3500 cm^-1^ and a more pronounced presence of C = C bonds compared to dental calculus. These features suggest greater biofilm resilience and higher inflammatory potential. Dental calculus exhibits sharper phosphate and carbonate peaks, reflecting higher mineral density and crystallinity, which may make it more brittle and easier to remove. These differences underscore the need for tailored debridement protocols and highlight the diagnostic value of FTIR in assessing peri-implant disease risk.

 These findings may reflect the influence of implant surface properties, such as titanium oxide layers and altered surface energy, on biofilm retention and maturation. Unlike enamel, implant surfaces lack salivary pellicle formation and exhibit distinct ion-adsorption dynamics, which may promote the accumulation of loosely bound organic material and delay crystallization.

 The broader O–H and C–H stretching bands observed in peri-implant samples suggest increased hydration and lipid content, potentially contributing to a softer, less crystalline deposit. This may have clinical implications for cleaning resistance: while dental calculus is typically more mineralized and tightly adherent, peri-implant calculus may be more biofilm-rich but less tenacious, possibly affecting the efficacy of mechanical debridement and ultrasonic scaling. However, the layered structure and the presence of microchannels in FESEM images could facilitate bacterial migration and recolonization, posing a challenge to long-term peri-implant health.

 The limitation of IR spectroscopy is that traditional spectrometers acquire IR spectra by analyzing the absorption of a monochromatic beam of radiation (single wavelength). The sample must be free of water, as water significantly absorbs radiation at wave numbers around 3700 cm^−1^ and 1630 cm^−1^, potentially obscuring the absorption bands of the substance being tested.^13^ In the ATR sampling system, a primary source of measurement errors arises from inadequate contact between the sample and the diamond crystal.^14^ Despite this limitation, ATR analysis has proved useful for indicating the main features in the process of crystallization of dental calculus.^14^ Additionally, a further limitation of FT-IR instruments is that they are typically designed with only a single beam, in contrast to dispersive instruments, which usually feature a double beam configuration.^15^

 FESEM imaging provided complementary morphological insights: while dental calculus showed denser microbial plaque accumulation, implant calculus exhibited a layered structure with multiple pores and reduced non-mineralized material on its surface. Taken together, these results indicate that variations in surface energy, mineral composition, and organic content collectively influence how calculus mineralizes and how vulnerable peri-implant surfaces may be to persistent biofilm and inflammation.

 The qualitative morphological assessment revealed that dental calculus exhibited densely packed bacterial imprints and limited porosity, consistent with mature biofilm calcification. In contrast, peri-implant calculus showed a more porous architecture with lamellar layering, which may reflect intermittent mineral deposition influenced by peri-implant inflammation or reduced salivary buffering capacity in the anterior mandible. These structural differences could also influence the local inflammatory response, as porous deposits may harbor anaerobic niches and resist complete removal during routine maintenance.

 Taken together, these findings support the hypothesis that peri-implant calculus differs not only in composition but also in its potential pathogenicity and response to treatment. While this study was descriptive and exploratory, future research incorporating quantitative imaging and longitudinal clinical data could clarify whether these differences translate into distinct disease trajectories or cleaning protocols.

 Future studies could compare supragingival and subgingival calculus on implants, as well as subgingival calculus on implants and teeth. Furthermore, comparative research conducted in other regions of the oral cavity may yield different outcomes, as this investigation was limited to the anterior mandible. Due to the limited amount of peri-implant calculus, elemental analysis and crystalline phase could not be evaluated, which could be assessed in future studies. Further research is essential to elucidate the clinical implications of these findings and their potential impact on oral health and implant longevity.

## Conclusion

 This study revealed distinct differences in the functional groups and surface morphology between peri-implant and dental calculus. FTIR revealed higher organic content in peri-implant samples, while dental calculus showed greater mineral crystallinity. FESEM imaging qualitatively demonstrated denser bacterial imprints and limited porosity in dental calculus (~0.5–1.0 µm), versus larger pores (~1–3 µm) and micro-channels (~0.4–0.7 µm) in peri-implant deposits. These differences suggest that peri-implant calculus may be more hydrated, less crystalline, and potentially more resistant to mechanical cleaning. Tailored maintenance strategies and further quantitative research are warranted to optimize peri-implant care.

## Competing Interests

 Currently, Behzad Houshmand serves on the Editorial Advisory Board of JAPID. The authors declare no other competing interests concerning authorship and/or publication of this article.

## Data Availability

 All data generated or analyzed during this study are included in this published article.

## Ethical Approval

 The protocol of this cross-sectional study was reviewed and approved by the Ethics Committee of Shahid Beheshti University of Medical Sciences, Tehran, Iran (IR.SBMU.DRC.REC.1402.029).
